# Supplementary material for: Plk1, upregulated by HIF-2, mediates metastasis and drug resistance of clear cell renal cell carcinoma
Source: Commun Biol. 2021 Feb 5;4:166. doi: 10.1038/s42003-021-01653-w (PMC7865059; doi:10.1038/s42003-021-01653-w)
Supplement: Supplementary file 2 — Supplementary Information [file 42003_2021_1653_MOESM2_ESM.pdf]

Supplementary Methods

Colony formation assay

ccRCC cells (500 cells per condition) were treated or not with sunitinib or volasertib. Colonies were detected after 10 days of culture. Cells were then washed, fixed and stained with GEMSA (Sigma).

Immunohistochemistry TMA

Samples were collected with the approval of the Local Ethics committee. Sections from blocks of formol-fixed and paraffin-embedded tissue were examined for immunostaining for Plk1. After deparaffinization, hydration and heat-induced antigen retrieval, the tissue sections were incubated for 20 min at room temperature with monoclonal anti-Plk1 antibody (Abcam, ab109777) diluted at 1:100. Biotinylated secondary antibody (DAKO) was applied and binding was detected with the substrate diaminobenzidine against a hematoxylin counterstain.

Neoadjuvant patients for qPCR analysis

Samples (tumor sections) were obtained from Nice, Bordeaux and Monaco hospitals. The patients' characteristics have already been described [1]. Patients were treated for at least two months before surgery (Supplementary Fig. 6a).

Gene expression microarray analysis

Normalised RNA sequencing (RNA-Seq) data produced by The Cancer Genome Atlas (TCGA) were downloaded from cBioportal (www.cbioportal.org, TCGA Provisional; RNA-Seq V2). Data were available for 503 of the 536 ccRCC tumor samples TCGA subjected to mRNA expression profiling. The subtype classifications were obtained through cBioPortal for Cancer Genomics and the 33 samples lacking classification were discarded. The non-metastatic group

contained 424 patients and the metastatic group contained 79 patients. The results published here are in whole or in part based upon data generated by the TCGA Research Network: http://cancergenome.nih.gov/[2,3] The Kaplan-Meier method was used to produce overall survival curves. The effect of Plk1 and its odds-ratio was estimated using a Cox model adjusted to the expression of other genes and important patient characteristics.

To evaluate the effect of Plk1 gene expression on ccRCC, we used the Kidney Renal Clear Cell Carcinoma (KIRC) dataset from The Cancer Genome Atlas (TCGA) [4]. RNA-seq and clinical data were downloaded from the TCGA data portal (https://portal.gdc.cancer.gov). RNA-seq data were normalised using the Bioconductor package DESeq2 and log2 transformed. The patients were separated into two groups with either a high or low Plk1 expression level (third quartile cut off). We then performed a differential analysis between the two groups of patients. P values were adjusted for multiple testing using the Benjamini and Hochberg procedure, which controls the false discovery rate (FDR).

We then performed a functional and pathways enrichment analysis on differentially expressed genes (FDR < 0.05 and absolute log2(Fold Change) > 1) based on KEGG, Gene Ontology and Reactome databases using the geneSCF tool [4]. The terms are considered significant only if enriched with a p value < 0.05.

References

1 Dufies, M. *et al.* Sunitinib Stimulates Expression of VEGFC by Tumor Cells and Promotes Lymphangiogenesis in Clear Cell Renal Cell Carcinomas. *Cancer Res* **77**, 1212-1226, doi:0008-5472.CAN-16-3088 [pii] 10.1158/0008-5472.CAN-16-3088 (2017).

2 Gao, J. *et al.* Integrative analysis of complex cancer genomics and clinical profiles using the cBioPortal. *Sci Signal* **6**, pl1, doi:scisignal.2004088 [pii] 10.1126/scisignal.2004088 (2013).

3 Cerami, E. *et al.* The cBio cancer genomics portal: an open platform for exploring multidimensional cancer genomics data. *Cancer Discov* **2**, 401-404, doi:2/5/401 [pii]10.1158/2159-8290.CD-12-0095 (2012).

4 Subhash, S. & Kanduri, C. GeneSCF: a real-time based functional enrichment tool with support for multiple organisms. *BMC Bioinformatics* **17**, 365, doi:10.1186/s12859-016-1250-z (2016).

Supplementary Figure Legends

Supplementary Table 1: High levels of Plk1 mRNA correlated to activation of the HIF pathway and to pejorative evolution of patients with different cancers.

a **HIF1 dependency**

Acute myeloid leukemia (165 patients)			
	**low Plk1**	**high Plk1**	**p value**
**PFS** (months)	ND	ND	
**OS** (months)	17	10	ns
	**high Ca9**	**high Oct4**	**high Glut1**
**Plk1 mRNA**	+	-/+	+
**p value**	0.041	ns	0.019

Adrenocortical (76 patients)			
	**low Plk1**	**high Plk1**	**p value**
**PFS** (months)	78	8	$4e^{-7}$
**OS** (months)	>160	18	$2e^{-13}$
	**high Ca9**	**high Oct4**	**high Glut1**
**Plk1 mRNA**	+	+	+
**p value**	0.05	ns	0.002

Bladder (402 patients)			
	**low Plk1**	**high Plk1**	**p value**
**PFS** (months)	43	22	0.017
**OS** (months)	38	27	ns
	**high Ca9**	**high Oct4**	**high Glut1**
**Plk1 mRNA**	+	-/+	+
**p value**	0.5	ns	0.045

**Cervical** (275 patients)			
	**low Plk1**	**high Plk1**	**p value**
**PFS** (months)	>200	>200	ns
**OS** (months)	95	>200	ns
	**high Ca9**	**high Oct4**	**high Glut1**
**Plk1 mRNA**	+	-/+	+
**p value**	0.002	ns	0.024

Colorectal (524 patients)			
	**low Plk1**	**high Plk1**	**p value**
**PFS** (months)	>140	>140	ns
**OS** (months)	66	>140	$5e^{-3}$
	**high Ca9**	**high Oct4**	**high Glut1**
**Plk1 mRNA**	+	+	+
**p value**	0.016	ns	0.001

Esophageal (181 patients)			
	**low Plk1**	**high Plk1**	**p value**
**PFS** (months)	21	18	ns
**OS** (months)	26	26	ns
	**high Ca9**	**high Oct4**	**high Glut1**
**Plk1 mRNA**	+	-/+	+
**p value**	0.04	ns	ns

**Glioblastoma** (145 patients)			
	**low Plk1**	**high Plk1**	**p value**
**PFS** (months)	6	8	ns
**OS** (months)	14	13	ns
	**high Ca9**	**high Oct4**	**high Glut1**
**Plk1 mRNA**	+	-/+	-/+
**p value**	0.04	ns	ns

HNSCC (552 patients)			
	**low Plk1**	**high Plk1**	**p value**
**PFS** (months)	76	4	0.056
**OS** (months)	65	32	0.003
	**high Ca9**	**high Oct4**	**high Glut1**
**Plk1 mRNA**	+	-/+	+
**p value**	<0.001	ns	ns

**Lung** (230 patients)			
	**low Plk1**	**high Plk1**	**p value**
**PFS** (months)	40	23	0.053
**OS** (months)	50	36	0.018
	**high Ca9**	**high Oct4**	**high Glut1**
**Plk1 mRNA**	+	-/+	+
**p value**	0.05	ns	<0.001

**Melanoma** (472 patients)			
	**low Plk1**	**high Plk1**	**p value**
**PFS** (months)	55	48	Ns
**OS** (months)	102	50	0.003
	**high Ca9**	**high Oct4**	**high Glut1**
**Plk1 mRNA**	+	-/+	+
**p value**	<0.001	ns	<0.001

**Pancreatic** (179 patients)			
	**low Plk1**	**high Plk1**	**p value**
**PFS** (months)	12	8	0.001
**OS** (months)	21	15	0.001
	**high Ca9**	**high Oct4**	**high Glut1**
**Plk1 mRNA**	+	-/+	+
**p value**	<0.001	ns	<0.001

Ovarian (201 patients)			
	**low Plk1**	**high Plk1**	**p value**
**PFS** (months)	16	18	ns
**OS** (months)	45	43	ns
	**high Ca9**	**high Oct4**	**high Glut1**
**Plk1 mRNA**	+	-/+	+
**p value**	0.05	ns	0.048

**chRCC** (66 patients)			
	**low Plk1**	**high Plk1**	**p value**
**PFS** (months)	>160	160	0.003
**OS** (months)	>160	>160	0.003
	**high Ca9**	**high Oct4**	**high Glut1**
**Plk1 mRNA**	+	-/+	+
**p value**	0.063	ns	0.013

**pRCC** (413 patients)			
	**low Plk1**	**high Plk1**	**p value**
**PFS** (months)	104	26	$2e^{-9}$
**OS** (months)	>160	58	$8e^{-10}$
	**high Ca9**	**high Oct4**	**high Glut1**
**Plk1 mRNA**	+	-/+	+
**p value**	0.001	ns	<0.001

Thyroid (480 patients)			
	**low Plk1**	**high Plk1**	**p value**
**PFS** (months)	>170	>170	$1e^{-3}$
**OS** (months)	>170	>170	ns
	**high Ca9**	**high Oct4**	**high Glut1**
**Plk1 mRNA**	+	-/+	+
**p value**	0.013	ns	0.05

b **HIF2 dependency**

**ccRCC** (413 patients)			
	**low Plk1**	**high Plk1**	**p value**
**PFS** (months)	124	33	$3e^{-9}$
**OS** (months)	>160	43	$2e^{-12}$
	**high Ca9**	**high Oct4**	**high Glut1**
**Plk1 mRNA**	-/+	+	+
**p value**	ns	0.007	<0.001

**Testicular** (144 patients)			
	**low Plk1**	**high Plk1**	**p value**
**PFS** (months)	>240	>240	ns
**OS** (months)	>240	229	ns
	**high Ca9**	**high Oct4**	**high Glut1**
**Plk1 mRNA**	-/+	+	+
**p value**	ns	<0.001	<0.001

c **HIF1 and HIF2 dependency**

**Breast** (960 patients)			
	**low Plk1**	**high Plk1**	**p value**
**PFS** (months)	>160	160	0.003
**OS** (months)	128	130	0.014
	**high Ca9**	**high Oct4**	**high Glut1**
**Plk1 mRNA**	+	+	+
**p value**	<0.001	<0.001	<0.001

**Glioma** (507 patients)			
	**low Plk1**	**high Plk1**	**p value**
**PFS** (months)	52	23	$9e^{-6}$
**OS** (months)	96	37	$5e^{-8}$
	**high Ca9**	**high Oct4**	**high Glut1**
**Plk1 mRNA**	+	+	+
**p value**	0.002	0.001	0.05

**Liver** (373 patients)			
	**low Plk1**	**high Plk1**	**p value**
**PFS** (months)	27	11	$3e^{-4}$
**OS** (months)	70	30	$1e^{-4}$
	**high Ca9**	**high Oct4**	**high Glut1**
**Plk1 mRNA**	+	+	+
**p value**	<0.001	<0.001	<0.001

**Mesothelioma** (82 patients)			
	**low Plk1**	**high Plk1**	**p value**
**PFS** (months)	16	7	$1e^{-3}$
**OS** (months)	24	6	$2e^{-8}$
	**high Ca9**	**high Oct4**	**high Glut1**
**Plk1 mRNA**	+	+	+
**p value**	ns	ns	0.028

**Prostate** (488 patients)			
	**low Plk1**	**high Plk1**	**p value**
**PFS** (months)	>160	67	$7e^{-8}$
**OS** (months)	>160	>160	ns
	**high Ca9**	**high Oct4**	**high Glut1**
**Plk1 mRNA**	+	+	+
**p value**	ns	ns	<0.001

**Sarcoma** (263 patients)			
	**low Plk1**	**high Plk1**	**p value**
**PFS** (months)	43	18	0.004
**OS** (months)	80	49	0.011
	**high Ca9**	**high Oct4**	**high Glut1**
**Plk1 mRNA**	+	+	+
**p value**	0.006	<0.001	<0.001

d **HIF1 or HIF2 independency**

**Stomach** (407 patients)			
	**low Plk1**	**high Plk1**	**p value**
**PFS** (months)	42	55	ns
**OS** (months)	29	36	ns
	**high Ca9**	**high Oct4**	**high Glut1**
**Plk1 mRNA**	+	-/+	+
**p value**	ns	ns	ns

**Uterine** (177 patients)			
	**low Plk1**	**high Plk1**	**p value**
**PFS** (months)	>160	> 160	0.047
**OS** (months)	>160	102	0.007
	**high Ca9**	**high Oct4**	**high Glut1**
**Plk1 mRNA**	-/+	-/+	-/+
**p value**	ns	ns	ns

**Uveal melanoma** (80 patients)			
	**low Plk1**	**high Plk1**	**p value**
**PFS** (months)	51	37	ns
**OS** (months)	46	37	ns
	**high Ca9**	**high Oct4**	**high Glut1**
**Plk1 mRNA**	+	-/+	-/+
**p value**	ns	ns	ns

PFS and OS were calculated from patient subgroups in the cBioportal database (TCGA PanCancer Atlas) with Plk1 mRNA levels that were less or greater than the third quartile. The total number of patients and the number of patients in each group are indicated. The levels of Plk1 mRNA (mRNA Expression z-Scores, RNA Seq V2 RSEM) are stratified into two groups expressing low or high levels of Ca9, Oct4 or Glut1. Ca9 served as a marker of HIF-1 activity, Oct4 served as a marker of HIF-2 activity and Glut1 served as a marker of HIF-1 and HIF-2 activities. Four groups related to Plk1 expression were defined: **a** HIF-1 dependency (Ca9 and Glut1); **b** HIF-2 dependency (Oct4 and Glut1), **c** HIF-1 and HIF-2 dependency (Ca9, Oct4 and Glut1); and **d** HIF-1 or HIF-2 independency (none of them).

Supplementary Fig. 1: Presence of a HRE in the *Plk1* promoter determined by *in silico* analysis.

cttaaattacaaaaattagccgggcatggtggtgcatgcttgtaattccagctgctagggaggccgaggcgggaggattgcttgaac
cctggaagcagaggttgcagtgagctgagatcgtgccactgcactccagcctgggcgacagagcaagattccgt**cacacacaca**aaa
aaaaggcgtgggggaggccaaacaaaaccccgcaagacacatttggctatgacctgccagtttgctaggcattcttccaaccttcc
ctccctctgaccaagaaactgagtgtccactattttaggccctgggaaattcagtagcgaggaggccagacagcttcgttgcatcat
ggggggctctggtactgtgcttctccaacttcaggatgtgtaggaatcacctgagcagtcttgttgagaggcggacactgactcggg
aggtctggggtagggcctgaacgtttgcctttgcggttctaacaagctctcaggtgatggcgatgctactgttccctggccccgagg
tagaggaagatttaagtaaaagattcctggaggaggcgcaagtgaaccgcaggagctttcccggacgcccgagaaagggagaaaccc
cgaaggaattcctcctctctcggggctgggtctccgcatccacgccgggtttggtttcccaggctatccc**acgtg**ttcgggcgtccg
tgtcaatcaggttttccccggctgggtccgggtttaaaggctgctgctgcgcagggcgctcccATGGTGCCGCGCGGCGGGCGGGTT
TGGATTTTAAATCCCCGCGGCCAATCAGTGGCGCGCAGGCTTTTGTAACGTTCCCAGCGCCGCGTTTGAATTCGGGGAGGAGCGGAG
CGGTGCGGAGGCTCTGCTCGGATCGAGGTCTGCAGCGCAGCTTCGGGAGC**ATG**AGTGCTGCAGTGACTGCAGGGAAGCTGG

HRE consensus sequence marked in bold.

Supplementary Fig. 2: Plk1 is associated with poor prognosis in ccRCC.

TCGA cohort - ccRCC patients

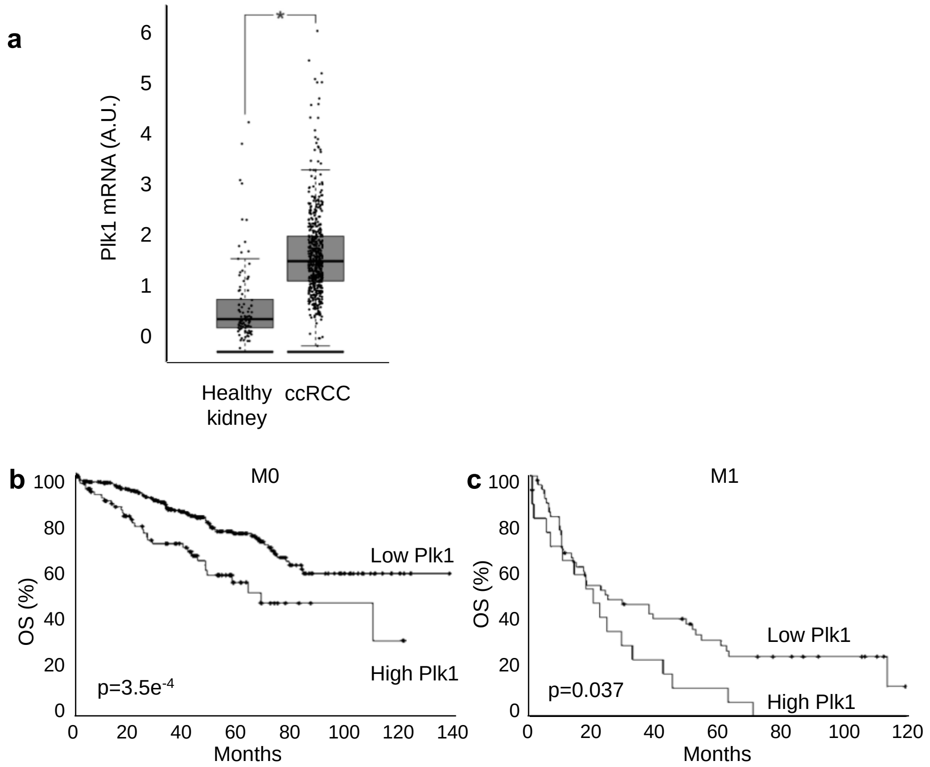

	Total cases	Median (month)
Low Plk1	283	<150
High Plk1	93	74

	Total cases	Median (month)
Low Plk1	53	26
High Plk1	17	21

a to c The tumors of ccRCC patients were analyzed for Plk1 mRNA levels (z-score). These results are in whole or in part based upon data generated by the TCGA Research Network. **A** Comparison of Plk1 mRNA levels between healthy kidney and ccRCC. **b and c** The levels of Plk1 mRNA in tumors of ccRCC patients M0 (**b**) or M1 (**c**) correlated with OS. OS were calculated from patient subgroups with mRNA levels that were less or greater than the third quartile value. Statistical significance (p value) is indicated.

Supplementary Table 2: The characteristics of the ccRCC patients included in TMA analysis (TMA cohort).

TMA cohort - ccRCC patients

**Number of patients**	131
age	60.2 (29-87)
**Gender**	
Female	43 (32.8%)
Male	88 (67.2%)
**Fuhrman grade**	
1	2 (1.5%)
2	58 (44.3%)
3	44 (33.6%)
4	27 (20.6%)
NA	
**pT**	
1	70 (53.4%)
2	18 (13.7%)
≥ 3	43 (32.8%)
**pN**	
0	118 (90.1%)
1	2 (1.5%)
2	9 (6.9%)
X	2 (1.5%)
**pM**	
0	101 (77.1%)
1	30 (22.9%)
**PFS (months) / progression %**	
M0	84.5 / 31.9%
M1	3.9 / 93.1%
**OS (months) / Death %**	
M0	NR / 17.6%
M1	16 / 93.1%

Patient characteristics included in Supplementary Fig. 3.

Supplementary Fig. 3: Detection of Plk1 on tumor sections by IHC (TMA cohort).

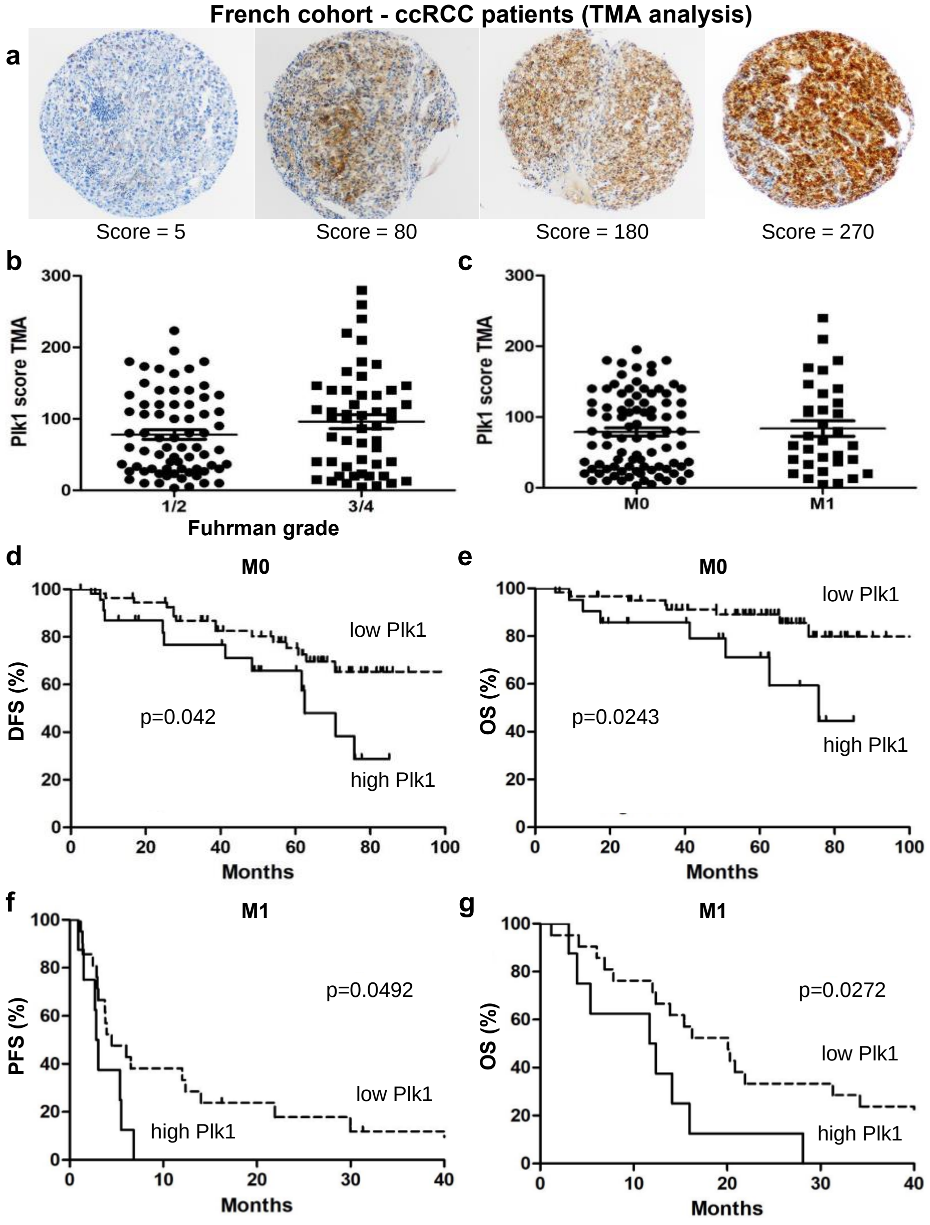

The percentage of cells and the intensity of the Plk1 labelling were evaluated. The Plk1 score was calculated form the percentage of labelled cells and the staining intensity. **a** Representatives images are shown. **b** The levels of the Plk1 score of ccRCC patients were compared to the Fuhrman grade 1/2 or 3/4. **c** The levels of the Plk1 score of M0 ccRCC patients and of M1 ccRCC patients were compared. **c and e** The levels of the Plk1 score of 101 M0 ccRCC patients correlated with DFS (**d**) or with OS (**e**). **f and g** The levels of the Plk1 score of 30 M1 ccRCC patients correlated with PFS (**f**) or with OS (**g**). The third quartile value of the Plk1 score (120) was chosen as the reference. The Kaplan-Meier method was used to produce survival curves and analyses of censored data were performed using Cox models. Statistical significance (p values) is indicated (see Supplementary Table 2).

Supplementary Fig. 4: HIF-2 bound directly to the Plk1 promoter and regulated its transcription.

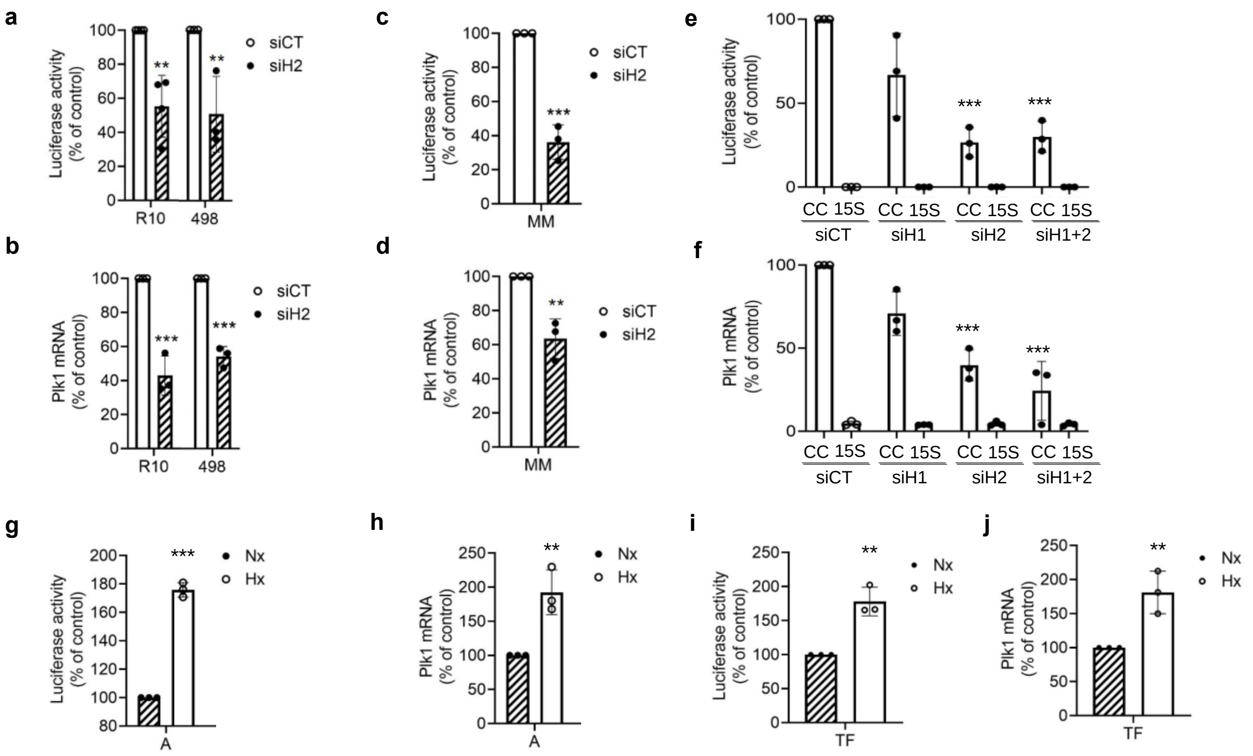

a to d ccRCC cell lines (VHL-inactivated) R10, 498 (**a, b**), or primary ccRCC cells (VHL-inactivated) MM (**c, d**) cells were transfected with siRNA against HIF-2α (H2) for 24 h. Cells were then transfected with a renilla luciferase reporter gene under the control of the Plk1 promoter. The renilla luciferase activity normalized to the firefly luciferase (control vector) represented the readout of the Plk1 promoter activity (**a, c**). The Plk1 mRNA level was determined by qPCR (**b, d**). **e and f** ccRCC primary ccRCC cells CC (VHL-inactivated) or healthy renal cells (15S) were transfected with siRNA against HIF-1□ (H1), or HIF-2□ (H2) or HIF-1□ and HIF-2□ (H1+2). 24 hours later, cells were transfected with a renilla luciferase reporter gene under the control of the Plk1 promoter. The renilla luciferase activity, normalized to the firefly luciferase (control vector), was a readout of the *Plk1* promoter activity (**e**). 48 hours after transfection, Plk1 mRNA levels were determined by qPCR (**f**). **g to j** RCC cell line (VHL-WT) A (**g, h**), or primary RCC cells (VHL-WT) TF (**i, j**) were cultured in normoxia (Nx) or hypoxia 1% O_2 (Hx) for 24 h. The renilla luciferase activity normalized to the firefly luciferase (control vector) represented the readout of the Plk1 promoter activity (**g, i**). The Plk1 mRNA level was determined by qPCR (**h, j**). Results are represented as the mean of three independent experiments ± SEM. Statistics were determined using an unpaired Student's *t* test: * p<0.05, ** p<0.01, *** p<0.001.

Supplementary Fig. 5: Analysis of pathway enrichment in ccRCC tumors according to the TCGA.

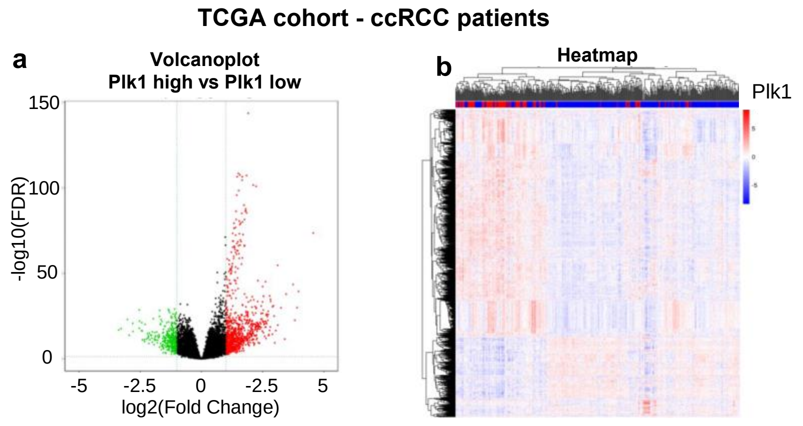

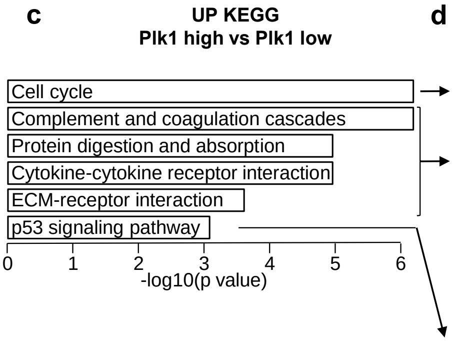

d

UP GO
Plk1 high vs Plk1 low

GO:	biological process	p value
**0051301**	cell division	$3.9e^{-09}$
**0008283**	cell proliferation	0.004
**0030198**	extracellular matrix organization	$2.5E^{-08}$
**0030335**	positive regulation of cell migration	0.001
**0060326**	cell chemotaxis	0.001
**0001837**	epithelial to mesenchymal transition	0.011
**1902042**	negative regulation of extrinsic apoptotic signaling pathway	0.031

The third quartile value of Plk1 expression was chosen as a cut-off. **a** Volcano plot showing the distribution of differentially expressed transcripts. 932 up-regulated genes in the Plk1 “high” group compared to the Plk1 “low” group are shown in red; 315 down-regulated genes are shown in green. Genes that were not differentially expressed (adj. p-value >0.05 and absolute log2(Fold change)>1) are shown in black. **b** Heatmap comparing the normalized log2 expression (z score) of the differentially expressed genes between the 110 patients with high Plk1 expression and the 328 patients with low Plk1 expression to obtain differentially expressed genes. **c** Graph of the top 6 enriched KEGG pathways from up-regulated genes. A Wilcoxon test was performed to obtain a p-value showing the differential significance. **d** Graph of the enriched GO pathways (link KEGG pathway) from up-regulated genes. A Wilcoxon test was performed to obtain a p-value showing the differential significance.

Supplementary Fig. 6: Sunitinib resistance are correlated with high Plk1 expression.

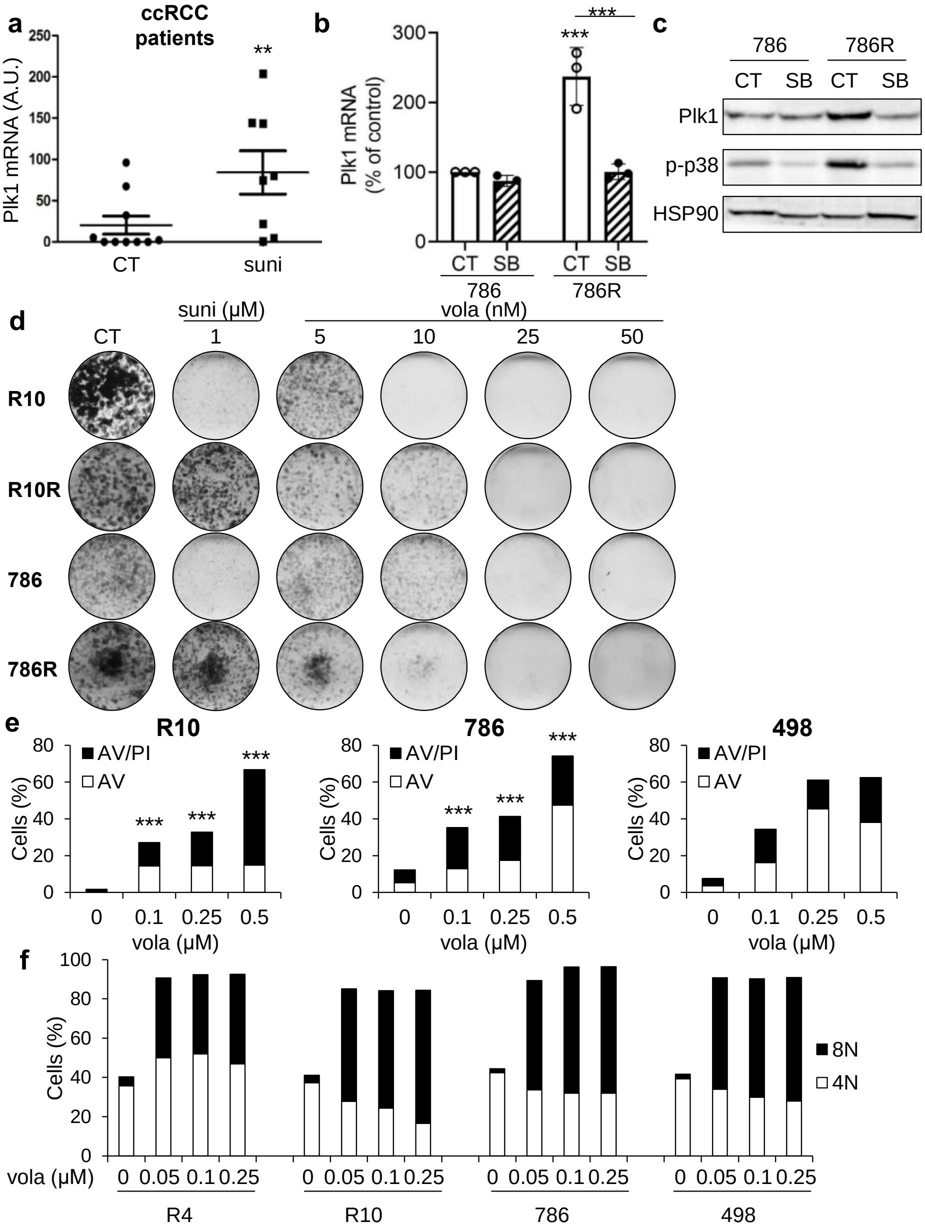

a The levels of Plk1 were determined by qPCR in tumors from patients either not treated (n=10) or treated with sunitinib in a neoadjuvant setting (n=10). **b and c** 786 and 786R cells were treated with 20 μM SB203580 (p38 inhibitor, SB) for 48 h. The Plk1 mRNA level was obtained by qPCR (**b**). Plk1, p-p38, p38 expression were evaluated by immunoblotting. HSP90 served as a loading control (**c**). **d** R10, R101R, 786 and 786R cells were incubated in the presence of volasertib (vola, 5 to 50 nM) or sunitinib (suni, 1 μM) and stained with Giemsa blue after 10 days. Results are representative of three independent experiments. **e and f** ccRCC cells were treated with volasertib (vola) for 48 h. **e** Cell death was evaluated by flow cytometry. Cells were stained with the PI/annexinV (AV). Histograms show AV^+/PI^- cells (apoptosis) and AV^+/PI^+ cells (post-apoptosis and/or others cell death). **f** Cells were labelled for 15 min with PI and analyzed by flow cytometry. Histograms represent the percentage of cells with a DNA content of 4N and 8N. Results are represented as means of three independent experiments ± SEM. Statistics were performed using an unpaired Student's *t* test: ** $p<0.01$, *** $p<0.001$.

Supplementary Table 3: The IC50 of the volasertib in different ccRCC cell lines, primary ccRCC cells and normal kidney cells.

Type of cells	Cell lines	IC50 Volasertib (nM)
ccRCC cell line	R4	55 +/- 6
	R10	85 +/- 10
	786	80 +/- 10
	498	90 +/- 8
Primary ccRCC cells	CC	260 +/- 13
	TF	750 +/- 14
	M	620 +/- 25
Normal kidney cells	14S	3 300 +/- 100
	15S	2 500 +/- 111
	18S	2 400 +/- 89

Cells were treated with volasertib for 48 h. Cell viability was measured with XTT assays and the IC50 was determined.

Supplementary Fig. 7: Volasertib induced polyploidy and apoptosis in ccRCC primary cells but not in normal cells.

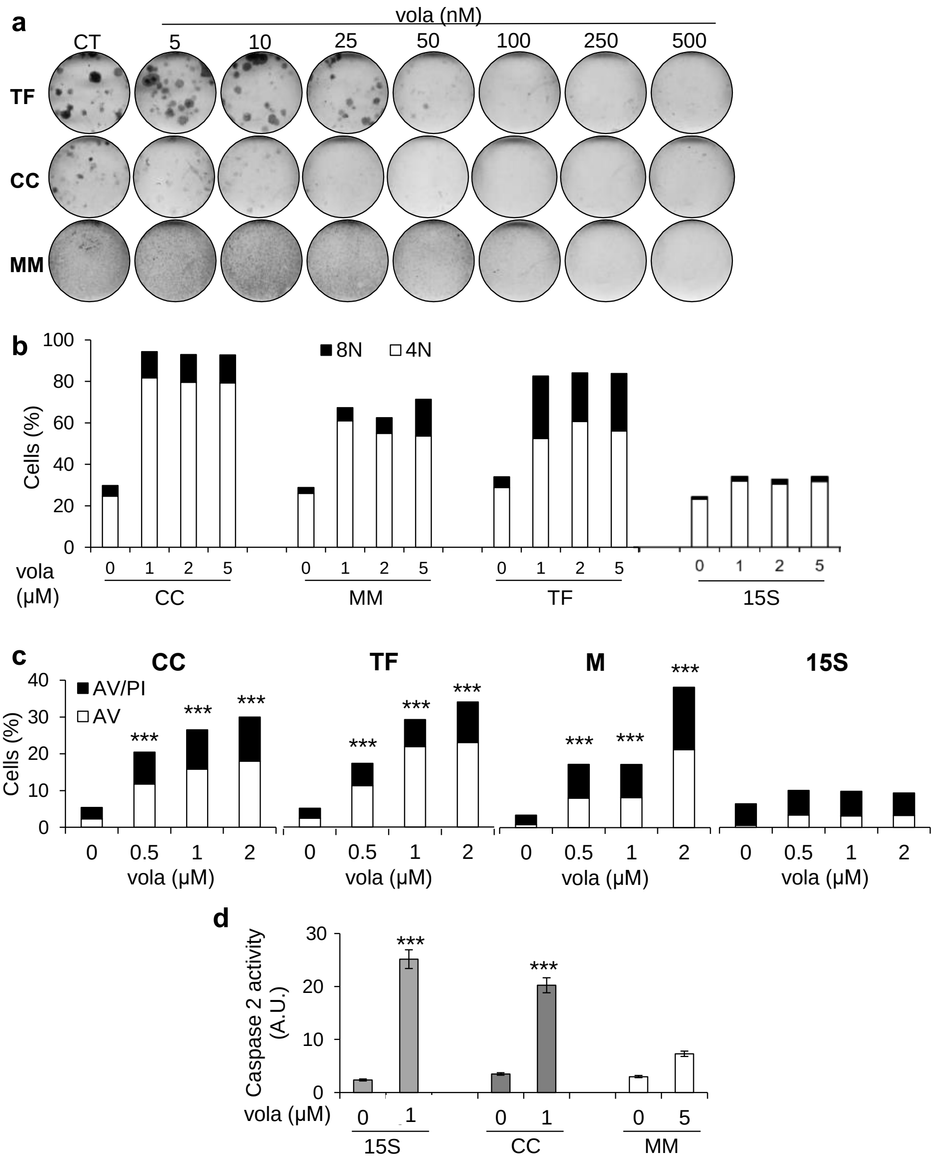

a Primary ccRCC cells were incubated in the presence of volasertib (vola, 1 to 500 nM) and stained with Giemsa blue after 10 days. Results are representative of three independent experiments. **b to d** Primary ccRCC cells and healthy renal cells (15S) were treated with volasertib (vola) for 48 h. Cells were labelled for 15 min with PI and analyzed by flow cytometry. Histograms represent the percentage of cells with a DNA content of 4N and 8N. Results are represented as means of three independent experiments (**b**). Cell death was evaluated by flow cytometry. Cells were stained with the PI/ AV. Histograms showed AV^+/PI^- cells (apoptosis) and AV^+/PI^+ cells (post-apoptosis and/or others cell death; **c**). The caspase 2 activity was evaluated using Ac-VDVAD-AMC as a substrate (**d**). Results are represented as means of three independent experiments ± SEM. Statistics were determined using an unpaired Student's *t* test: * p<0.05, *** p<0.001.

Supplementary Fig. 8: Volasertib inhibited the growth of experimental ccRCC and induced necrosis in 3D ccRCC primary tumors.

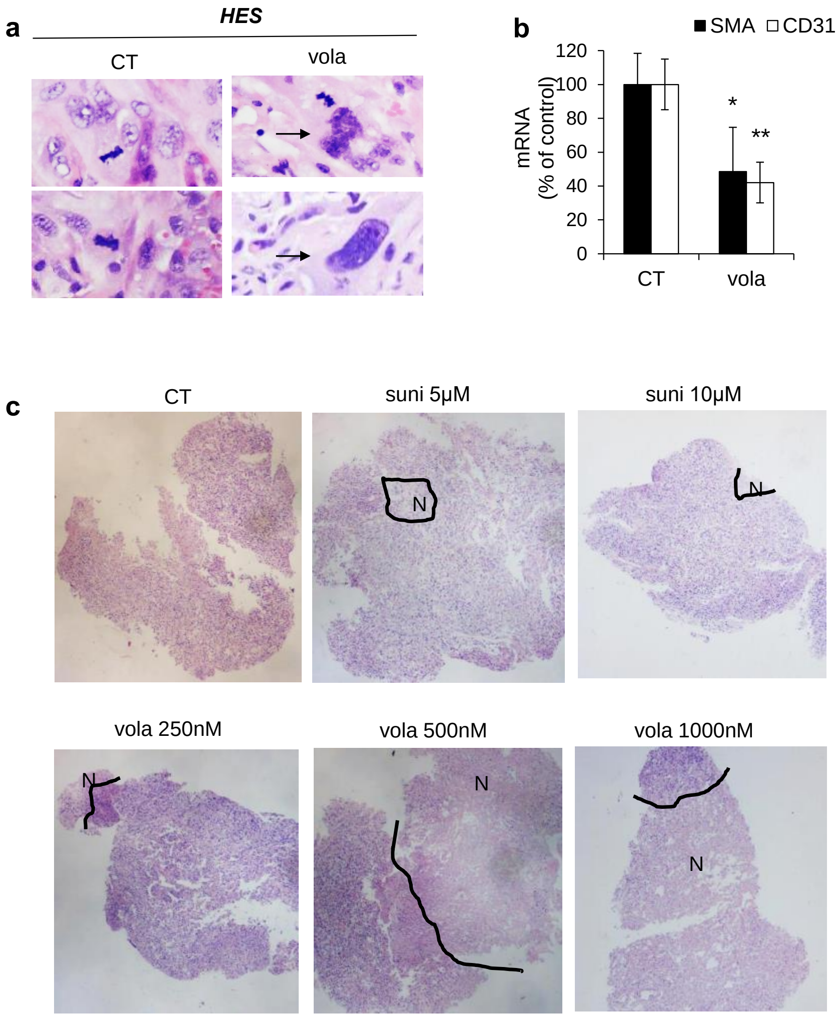

a to b 7.10[6] 786-O cells were subcutaneously injected into the flank of nude mice (*n*=8 per group). 30 days after injection, all mice developed tumors and were treated with a control solution or 20 mg/kg volasertib by gavage twice a week. **a** HES coloration. Representative images are shown. Arrows indicate giant cells. **b** The mRNA levels of CD31 and αSMA in tumors were determined by qPCR.One-way ANOVA was used for statistical comparison: * p<0.05, *** p<0.001. **c** Representatives images of HES staining of 3D primary ccRCC tumors treated 72h with sunitinib (suni) or volasertib (vola). N: necrosis

Supplementary Fig. 9: *In silico* analysis of TCGA and TCIA databases.

TCGA cohort - Metastatic ccRCC patients

a

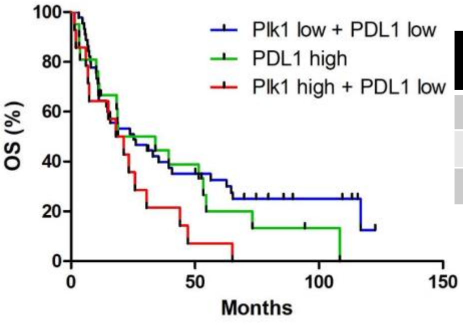

p=0.041	Total cases	Median (month)
Plk1 low + PDL1 low	44	25.2
PDL1 high	22	26.5
Plk1 high + PDL1 low	14	19.7

b

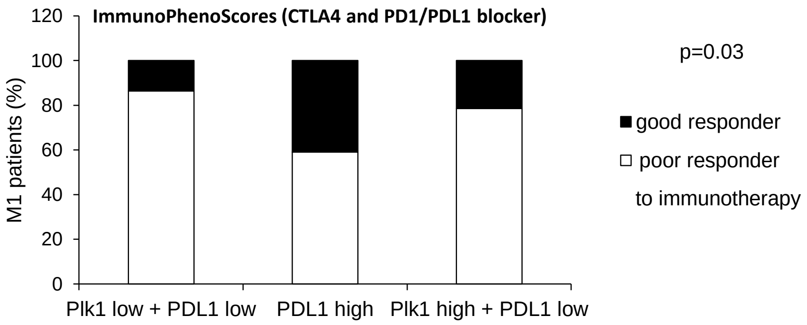

These results are in whole or in part based upon data generated by the TCGA Research Network. **a** Analysis of the cBioportal database, the levels of Plk1 and PDL1 mRNA in 80 metastatic (M1) ccRCC patients correlated with OS. **b** Analysis of the TCIA database, the levels of Plk1 and PDL1 mRNA in 80 metastatic (M1) ccRCC patients correlated with the immunophenoscore (IPS, score predicting the response to CTLA4 and PD1/PDL1 immunotherapy). An IPS between 5 and 8 corresponded to poor immunotherapy responder patients, and an IPS between 9 and 10 corresponded to good responder immunotherapy patients. **c** The third quartile value of Plk1 and PDL1 expression was chosen as the reference. The Kaplan-Meier method was used to produce survival curves and analyses of censored data were performed using Cox models. Statistical significance (p values) is indicated.

Supplementary Unprocessed westernblots

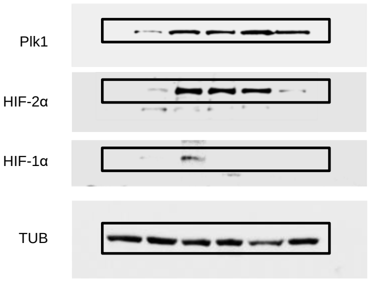

Fig. 2a

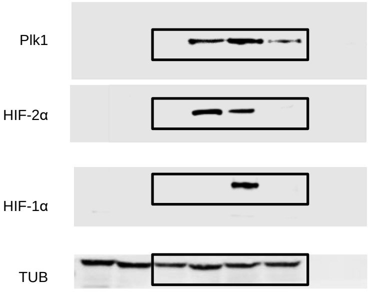

Fig. 2b

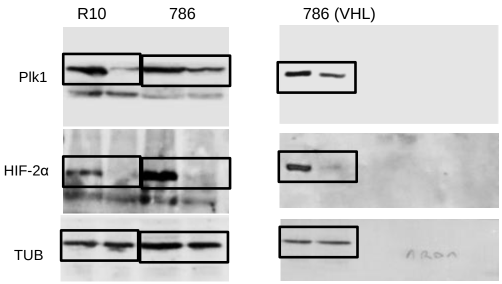

Fig. 2f

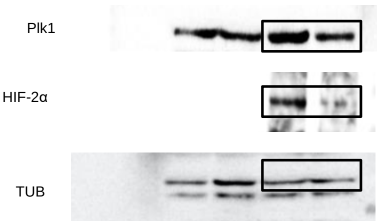

Fig. 2g

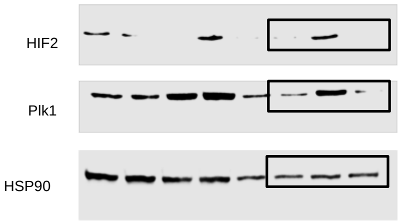

Fig. 2h

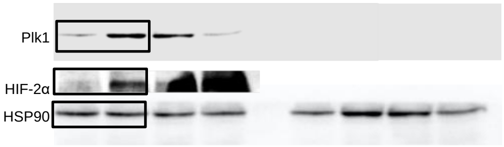

Fig. 2i

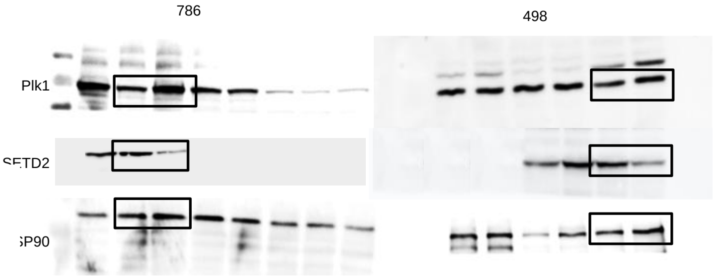

Fig. 3e

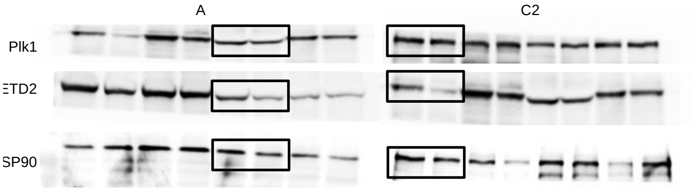

Fig. 3f

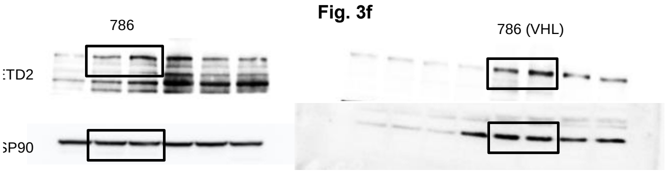

Fig. 3i

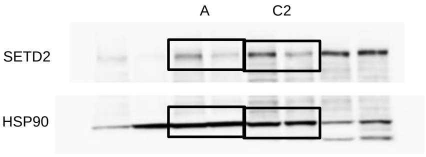

Fig. 3j

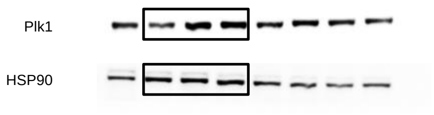

Fig. 4a

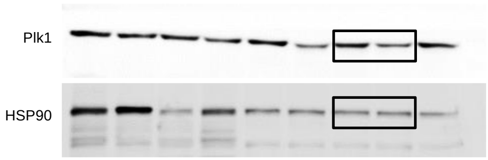

Fig. 4g

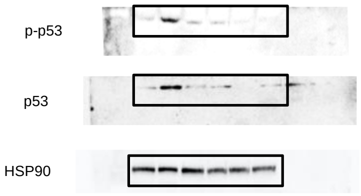

Fig. 4e
