# Supplementary material for: Plk1, upregulated by HIF-2, mediates metastasis and drug resistance of clear cell renal cell carcinoma
Source: Commun Biol. 2021 Feb 5;4:166. doi: 10.1038/s42003-021-01653-w (PMC7865059; doi:10.1038/s42003-021-01653-w)
Supplement: Supplementary file 3 — Description of Additional Supplementary Files [file 42003_2021_1653_MOESM3_ESM.pdf]

Description of Additional Supplementary Files

File name: Supplementary Data 1

Description: Source data underlying the graphs and charts.
