# Supplementary material for: Plk1, upregulated by HIF-2, mediates metastasis and drug resistance of clear cell renal cell carcinoma
Source: Commun Biol. 2021 Feb 5;4:166. doi: 10.1038/s42003-021-01653-w (PMC7865059; doi:10.1038/s42003-021-01653-w)
Supplement: Supplementary file 4 — Supplementary Data 1 [file 42003_2021_1653_MOESM4_ESM.docx]

**Figure 2D**

| siCT | | | | | siH2 | | | | |
| --- | --- | --- | --- | --- | --- | --- | --- | --- | --- |
| 100 | 100 | 100 | 100 | 100 | 44,8 | 44,9 | 17,3 | 59,1 | 56,4 |

**Figure 2E**

|  | siCT | | | | | siH2 | | | | |
| --- | --- | --- | --- | --- | --- | --- | --- | --- | --- | --- |
| 786 | 100 | 100 | 100 | 100 | 100 | 58,6 | 45,3 | 4,8 | 21,1 | 39,06 |

**Figure 2F**

|  | siCT | | | | siH2 / VHL | | | |
| --- | --- | --- | --- | --- | --- | --- | --- | --- |
| R10 | 100 | 100 | 100 |  | 30,97656 | 50,6 | 40,4580153 | 57,2519084 |
| 498 | 100 | 100 | 100 |  | 25,5102041 | 45,855 | 35,255102 |  |
| 786 | 100 | 100 | 100 |  | 40,2684564 | 35,8974359 | 38,0829461 | 13,7813669 |
| 786 | 100 | 100 | 100 |  | 76,8953069 | 68,6025408 | 85,0865 |  |

| **Figure 2H** | **NX**  **siCT** | | | | HX  siCT | | | | HX  siH2 | | | |
| --- | --- | --- | --- | --- | --- | --- | --- | --- | --- | --- | --- | --- |
| A | 100 | 100 | 100 |  | 264 | 203 | 183,9 | 225,3 | 80 | 118 | 129 |  |

**Figure 2I**

|  | Nx | | | | Hx | | | |
| --- | --- | --- | --- | --- | --- | --- | --- | --- |
| TF | 100 | 100 | 100 |  | 183 | 212 | 151 |  |

**Figure 3C**

|  | siCT | | | | siS2 | | | |
| --- | --- | --- | --- | --- | --- | --- | --- | --- |
| 786 | 100 | 100 | 100 | 100 | 170,8725 | 170,3628 | 214,3267 | 154,037 |
| 498 | 100 | 100 | 100 | 100 | 91,70467 | 78,53761 | 104,0781 | 108,6 |

**Figure 3D**

|  | siCT | | | | siS2 | | | |
| --- | --- | --- | --- | --- | --- | --- | --- | --- |
| A | 100 | 100 | 100 |  | 64,95819 | 84,95819 | 88,18237 |  |
| C2 | 100 | 100 | 100 |  | 91,70467 | 78,53761 | 94,0781 |  |

|  | siCT | | | | siS2 | | | |
| --- | --- | --- | --- | --- | --- | --- | --- | --- |
| A | 100 | 100 | 100 |  | 41,07555 | 11,70467 | 16,60413 |  |
| C2 | 100 | 100 | 100 |  | 31,67 | 28,25961 | 14,129 |  |

**Figure 3E**

|  | siCT | | | | siS2 | | | |
| --- | --- | --- | --- | --- | --- | --- | --- | --- |
| 786 | 100 | 100 | 100 |  | 214 | 197 | 221 |  |
| 498 | 100 | 100 | 100 |  | 106 | 93 | 101 |  |

**Figure 3F**

|  | siCT | | | | siS2 | | | |
| --- | --- | --- | --- | --- | --- | --- | --- | --- |
| A | 100 | 100 | 100 |  | 89 | 103 | 79 |  |
| C2 | 100 | 100 | 100 |  | 104 | 96 | 97 |  |

**Figure 3G**

| siCT | | | | siH2 | | | | CT | | | | VHL | | |
| --- | --- | --- | --- | --- | --- | --- | --- | --- | --- | --- | --- | --- | --- | --- |
| 100 | 100 | 100 | 100 | 177,4 | 140,9 | 135,2 | 141,4 | 100 | 100 | 100 | 100 | 227 | 213 | 178 |

**Figure 3H**

|  | Nx | | | | Hx | | | |
| --- | --- | --- | --- | --- | --- | --- | --- | --- |
| A | 100 | 100 | 100 |  | 70 | 63,05695 | 60,36792 |  |
| C2 | 100 | 100 | 100 |  | 72,53731 | 86,5887 | 79,46002 |  |

**Figure 3I**

| siCT | | | siH2 | | | CT | | | | VHL | | |
| --- | --- | --- | --- | --- | --- | --- | --- | --- | --- | --- | --- | --- |
| 100 | 100 | 100 | 225 | 201 | 239 | 100 | 100 | 100 | 100 | 342 | 283 | 310 |

**Figure 3J**

|  | Nx | | | | Hx | | | |
| --- | --- | --- | --- | --- | --- | --- | --- | --- |
| A | 100 | 100 | 100 |  | 70 | 53 | 50 |  |
| C2 | 100 | 100 | 100 |  | 89 | 69 | 76 |  |

**Figure 4A**

| 786 EV | | | | 786  *plk1-1* | | | | 786 *plk1-2* | | | |
| --- | --- | --- | --- | --- | --- | --- | --- | --- | --- | --- | --- |
| 100 | 100 | 100 |  | 230 | 261 | 253 |  | 311 | 253 | 284 |  |

**Figure 4B**

| 786 EV | | | | 786  *plk1-1* | | | | 786 *plk1-2* | | | |
| --- | --- | --- | --- | --- | --- | --- | --- | --- | --- | --- | --- |
| 100 | 100 | 100 | 100 | 352 | 302 | 329 | 289 | 193 | 248 | 261 | 189 |

**Figure 4C**

|  | 786 EV | | | | 786  *plk1-1* | | | | 786 *plk1-2* | | | |
| --- | --- | --- | --- | --- | --- | --- | --- | --- | --- | --- | --- | --- |
| 0 | 100 | 100 | 100 |  | 100 | 100 | 100 |  | 100 | 100 | 100 |  |
| 2.5 | 62 | 51 | 54 |  | 80 | 75 | 79 |  | 75 | 69 | 82 |  |
| 5 | 10 | 11 | 20 |  | 52 | 38 | 54 |  | 51 | 59 | 56 |  |

**Figure 4D**

|  | 786 EV | | | | 786  *plk1-1* | | | | 786 *plk1-2* | | | |
| --- | --- | --- | --- | --- | --- | --- | --- | --- | --- | --- | --- | --- |
| 0 | 9 | 3 | 8 |  | 5 | 7 | 6 |  | 8 | 3 | 7 |  |
| 2.5 | 18 | 22 | 29 |  | 7 | 11 | 12 |  | 10 | 8 | 14 |  |
| 5 | 48 | 55 | 40 |  | 25 | 26 | 21 |  | 17 | 22 | 13 |  |

**Figure 4G**

|  | 786 | | | | 786R | | | |
| --- | --- | --- | --- | --- | --- | --- | --- | --- |
| 786 | 100 | 100 | 100 | 100 | 190 | 259 | 296 | 216 |

Figure 5A

| days | 30 | 34 | 37 | 39 | 41 | 44 | 46 | 48 | 51 | 54 |
| --- | --- | --- | --- | --- | --- | --- | --- | --- | --- | --- |
| CT | 44 | 56 | 83 | 100 | 185 | 220 | 299 | 348 | 436 | 731 |
| vola | 45 | 57 | 50 | 52 | 66 | 82 | 105 | 118 | 132 | 140 |
| suni | 44 | 65 | 80 | 94 | 107 | 140 | 196 | 215 | 283 | 317 |

**Figure 5C**

| CT | 21 | 18 | 19 | 21 | 14 | 21 | 22 | 24 | 30 | 23 | 19 | 23 | 24 | 21 | 22 |
| --- | --- | --- | --- | --- | --- | --- | --- | --- | --- | --- | --- | --- | --- | --- | --- |
| Vola | 14 | 16 | 12 | 12 | 10 | 13 | 14 | 10 | 6 | 8 | 6 | 16 | 20 | 21 | 12 |

**Figure 5E**

| CT | Vola |
| --- | --- |
| 1,49675 | 1,46575 |
| 1,65 | 0,898 |
| 2,4545 | 1,2905 |
| 1,53075 | 1,377667 |
| 1,8 | 0,99175 |
| 1,391 | 1,505 |

**Figure 5I**

| CT | S5 | S10 | v100 | v250 | v500 | v1000 |
| --- | --- | --- | --- | --- | --- | --- |
| 100 | 71,25444996 | 52,2683375 | 93,2462793 | 53,24745282 | 41,60496574 | 14,5790462 |

**Figure 5J**

| CT | 7 | 0 | 5 | 14 |
| --- | --- | --- | --- | --- |
| s5 | 8 | 4 | 7 | 24 |
| s10 | 24 | 11 | 30 | 63 |
| v100 | 10 | 0 | 48 | 12 |
| v250 | 25 | 8 | 49 | 20 |
| v500 | 57 | 62 | 48 | 81 |
| v1000 | 76 | 88 | 71 | 92 |
