# Supplementary material for: Plk1, upregulated by HIF-2, mediates metastasis and drug resistance of clear cell renal cell carcinoma
Source: Commun Biol. 2021 Feb 5;4:166. doi: 10.1038/s42003-021-01653-w (PMC7865059; doi:10.1038/s42003-021-01653-w)
Supplement: Supplementary file 5 — Reporting Summary [file 42003_2021_1653_MOESM5_ESM.pdf]

nature research

Corresponding author(s): Maeva Dufies et Gilles Pagès

Last updated by author(s): Nov 3, 2020

Reporting Summary

Nature Research wishes to improve the reproducibility of the work that we publish. This form provides structure for consistency and transparency in reporting. For further information on Nature Research policies, see our Editorial Policies and the Editorial Policy Checklist.

Statistics

For all statistical analyses, confirm that the following items are present in the figure legend, table legend, main text, or Methods section.

n/a	Confirmed	
☐	☒	The exact sample size (n) for each experimental group/condition, given as a discrete number and unit of measurement
☐	☒	A statement on whether measurements were taken from distinct samples or whether the same sample was measured repeatedly
☐	☒	The statistical test(s) used AND whether they are one- or two-sided<br>*Only common tests should be described solely by name; describe more complex techniques in the Methods section.*
☐	☒	A description of all covariates tested
☐	☒	A description of any assumptions or corrections, such as tests of normality and adjustment for multiple comparisons
☐	☒	A full description of the statistical parameters including central tendency (e.g. means) or other basic estimates (e.g. regression coefficient) AND variation (e.g. standard deviation) or associated estimates of uncertainty (e.g. confidence intervals)
☐	☒	For null hypothesis testing, the test statistic (e.g. F, t, r) with confidence intervals, effect sizes, degrees of freedom and P value noted<br>*Give P values as exact values whenever suitable.*
☒	☐	For Bayesian analysis, information on the choice of priors and Markov chain Monte Carlo settings
☐	☒	For hierarchical and complex designs, identification of the appropriate level for tests and full reporting of outcomes
☐	☒	Estimates of effect sizes (e.g. Cohen's d, Pearson's r), indicating how they were calculated

Our web collection on statistics for biologists contains articles on many of the points above.

Software and code

Policy information about availability of computer code

Data collection	R software, Excel, GraphPad Prism
Data analysis	R software, Excel, GraphPad Prism

For manuscripts utilizing custom algorithms or software that are central to the research but not yet described in published literature, software must be made available to editors and reviewers. We strongly encourage code deposition in a community repository (e.g. GitHub). See the Nature Research guidelines for submitting code & software for further information.

Data

Policy information about availability of data

All manuscripts must include a data availability statement. This statement should provide the following information, where applicable:

- Accession codes, unique identifiers, or web links for publicly available datasets
- A list of figures that have associated raw data
- A description of any restrictions on data availability

data are avaible if ask maeva.dufies@gmail.com

Field-specific reporting

Life sciences study design

All studies must disclose on these points even when the disclosure is negative.

Sample size	For in vitro and in vivo analysis<br>Results are represented as means of three or more independent experiments (biological replication). All data are expressed as the mean ± the standard error (SEM). Statistical significance and p values were determined by the two-tailed Student's t-test. One-way ANOVA was used for statistical comparisons. Data were analyzed with Prism 5.0b (GraphPad Software) by one-way ANOVA with Bonferroni post hoc.<br>For patients<br>The Student's t-test was used to compare continuous variables and chi-square test, or Fisher's exact test (when the conditions for use of the $\chi 2$-test were not fulfilled), were used for categorical variables. To guarantee the independence of Plk1 as a prognostic factor, the multivariate analysis was performed using a Cox regression adjusted to the Fuhrman grade. DFS was defined as the time from surgery to the appearance of metastasis. PFS was defined as the time between surgery and progression, or death from any cause, censoring live patients and progression free at the last follow-up. OS was defined as the time between surgery and the date of death from any cause, censoring those alive at the last follow-up. The Kaplan-Meier method was used to produce survival curves and analyses of censored data were performed using Cox models. All analyses were performed using R software, version 3.2.2 (Vienna, Austria, https://www.r-project.org/).
Data exclusions	no data exclusion
Replication	Results are represented as means of three or more independent experiments (biological replication)
Randomization	NA
Blinding	NA

Reporting for specific materials, systems and methods

We require information from authors about some types of materials, experimental systems and methods used in many studies. Here, indicate whether each material, system or method listed is relevant to your study. If you are not sure if a list item applies to your research, read the appropriate section before selecting a response.

Materials & experimental systems

n/a	Involved in the study	
☐	☒	Antibodies
☐	☒	Eukaryotic cell lines
☒	☐	Palaeontology and archaeology
☐	☒	Animals and other organisms
☐	☒	Human research participants
☐	☒	Clinical data
☒	☐	Dual use research of concern

Methods

n/a	Involved in the study	
☒	☐	ChIP-seq
☐	☒	Flow cytometry
☒	☐	MRI-based neuroimaging

Antibodies

Antibodies used	Anti-HSP90 and anti-tubulin antibodies were purchased from Santa Cruz Biotechnology. Anti-Plk1 antibodies were purchased from Abcam. The anti-HIF-2α antibody was purchased from Novus Biochemicals. The rabbit polyclonal anti-HIF-1α antibodies were previously described (Richard, D. E., Berra, E., Gothie, E., Roux, D. & Pouyssegur, J. p42/p44 mitogen-activated protein kinases phosphorylate hypoxia-inducible factor 1alpha (HIF-1alpha) and enhance the transcriptional activity of HIF-1. J Biol Chem 274, 32631-32637. (1999).)
Validation	validation by manufacter and/or publication

Eukaryotic cell lines

Policy information about cell lines

Cell line source(s)	Different kidney cancer cell lines and primary cells were used in this study, including clear cell RCC (ccRCC) cell lines (caki-2, RCC4, RCC10, 786-O) and primary ccRCC cells (CC, MM), but also other type of RCC including papillary (pRCC) cell lines (A-498, ACHN) and primary RCC cells with a TFE3 translocation (TF). RCC4 (R4), ACHN (A), Caki-2 (C2), 786-0 (786) and A498 (498) RCC cell lines were purchased from the American Tissue Culture Collection. RCC10 (R10) was a kind gift from Dr. W.H. Kaelin (Dana-Farber Cancer Institute, Boston, MA). Primary cells were already described and cultured in a medium specific for renal cells (PromoCell, Heidelberg Germany) 19. 786R and RCC10R were previously described 36.
Authentication	ATCC
Mycoplasma contamination	tested negative for mycoplasm comtamination

Commonly misidentified lines (See ICLAC register)	no

Animals and other organisms

Policy information about studies involving animals; ARRIVE guidelines recommended for reporting animal research

Laboratory animals	Zebrafish and nude (nu/nu) mice
Wild animals	Zebrafish embryos were raised at 28°C under standard experimental conditions. At the age of 24 hpf, they were incubated in aquarium water containing 0.2 mmol/L 1-phenyl-2-thio-urea (PTU, Sigma). At 48-hpf, they were dechorionated with a pair of sharp-tip forceps and anesthetized with 0.04 mg/mL of tricaine (MS-222, Sigma). Anesthetized embryos were subjected for microinjection. 7. 106 786 cells were injected subcutaneously into the flank of 5-week-old nude (nu/nu) female mice (Janvier, France). The tumor volume was determined with a caliper (v = L*l2*0.5). When the tumor reached 50 mm3, mice were treated five times a week for 4 weeks, by gavage with placebo (dextrose water vehicle), sunitinib (40 mg/kg) or twice a week for 4 weeks with volasertib (25 mg/kg).
Field-collected samples	Mice tumor were immediatly put at -80°C and/or fixated with paraformaldehyde before paraffin-embedded
Ethics oversight	Zebrafish : All animal experiments were approved by the Northern Stockholm Experimental Animal Ethical Committee.<br>Mice: This study was carried out in strict accordance with the recommendations of the Guide for the Care and Use of Laboratory Animals. Our experiments were approved by the ‘‘Comité national institutionnel d'éthique pour l'animal de laboratoire (CIEPAL)'' (reference: NCE/2015-255).

Note that full information on the approval of the study protocol must also be provided in the manuscript.

Human research participants

Policy information about studies involving human research participants

Population characteristics	yes, see table 1, table 2 and Supplementary Table 2
Recruitment	no selection of patients
Ethics oversight	Informed consent was obtained from all individual participants included in the study. All patients gave written consent for the use of tumor samples for research. The study included only the major patients. This study was conducted in accordance with the Declaration of Helsinki.<br>From France and Belgium Hospital

Note that full information on the approval of the study protocol must also be provided in the manuscript.

Clinical data

Policy information about clinical studies

All manuscripts should comply with the ICMJEguidelines for publication of clinical research and a completedCONSORT checklist must be included with all submissions.

Clinical trial registration	na
Study protocol	na
Data collection	local hospital data collection
Outcomes	DFS was defined as the time from surgery to the appearance of metastasis. PFS was defined as the time between surgery and progression, or death from any cause, censoring live patients and progression free at the last follow-up. OS was defined as the time between surgery and the date of death from any cause, censoring those alive at the last follow-up.

Flow Cytometry

Plots

Confirm that:

- [x] The axis labels state the marker and fluorochrome used (e.g. CD4-FITC).
- [x] The axis scales are clearly visible. Include numbers along axes only for bottom left plot of group (a 'group' is an analysis of identical markers).
- [ ] All plots are contour plots with outliers or pseudocolor plots.
- [x] A numerical value for number of cells or percentage (with statistics) is provided.

Methodology

Sample preparation	Analysis of apoptosis<br>After stimulation, cells were washed with ice-cold PBS and stained with the annexin-V-fluos staining kit (Roche) according to

the manufacturer's procedure.
Cell cycle analysis
After treatment, cells were washed, fixed in ethanol 70% and, finally, left overnight at -20°C. Cells were next incubated in PBS, 3 µg/ml RNase A and 40 µg/ml of propidium iodide (PI) for 30 min at 4 °C.

Instrument	FACS-Calibur cytometer
Software	FACS-Calibur cytometer software
Cell population abundance	at least 10 000 cells per condition
Gating strategy	negative controle is used to make gates

☐ Tick this box to confirm that a figure exemplifying the gating strategy is provided in the Supplementary Information.
